# Should We Still Feel Reluctant About Prescribing Rivaroxaban for Advanced Chronic Kidney Disease?∗

**DOI:** 10.1016/j.jacadv.2023.100812

**Published:** 2024-01-04

**Authors:** George Ntaios, Dimitrios Sagris, Anastasia Adamou

**Affiliations:** Faculty of Medicine, Department of Internal Medicine, School of Health Sciences, University of Thessaly, Larissa, Thessaly, Greece

**Keywords:** atrial fibrillation, direct oral anticoagulants, renal disease, vitamin K agonists

**Figure:**
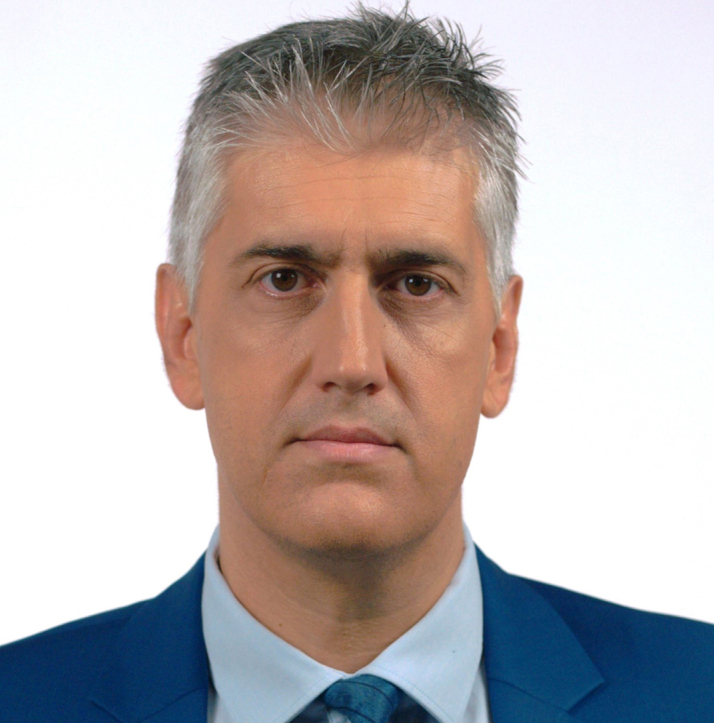

Atrial fibrillation (AF) and chronic kidney disease (CKD) frequently coexist, as almost one in 6 persons with CKD simultaneously has AF.^1^ Nevertheless, an important proportion of AF patients, especially those with estimated glomerular filtration rate <30 ml/min/1.73 m^2^, were either excluded or underrepresented in the major randomized clinical trials of direct oral anticoagulants (DOACs),^2^, ^3^, ^4^, ^5^ and data on their use in persons with severe CKD are extracted mainly from real-world observational studies.^6^ Furthermore, the current guidelines of American Heart Association/American College of Cardiology/Heart Rhythm Society for the management of persons with AF propose that treatment with reduced doses of direct thrombin or factor Xa inhibitors may be considered in persons with AF and moderate-to-severe CKD with an Class IIb of recommendation with a Level of Evidence: B-R.^7^ The lack of solid data on the safety and effectiveness of DOACs in severe CKD frequently leads to the hesitation of physicians to prescribe DOAC in AF patients with severe CKD, pointing toward an individualized approach especially in elderly and frail patients.^8^

As part of the holistic approach in the treatment of AF patients, protection against end-organ damage such as the brain and kidneys is essential. Previous studies have suggested a favorable effect of anticoagulation, especially with DOACs, on the progression of dementia.^9^ In the same context, better adherence to anticoagulation, especially with DOACs, was associated with a lower risk of acute kidney injury among patients with AF.^10^ Despite their important anticoagulation effect, vitamin K agonists’ (VKAs) were associated with damage to the vascular substrate of the kidneys when the international normalized ratio was elevated.^11^ On the other hand, the VKAs have the advantage that their elimination is performed by the liver.^12^

In this issue of *JACC: Advances*, Kreutz et al^13^ presented the results of the large prospective observational clinical study XARENO, which assessed the effect of rivaroxaban compared to VKAs in persons with AF and advanced CKD. The study included the underrepresented category of patients with estimated glomerular filtration rate 15 to 45 ml/min/1.73 m^2^, and after propensity score matching, it showed that rivaroxaban was associated with a significantly lower risk of adverse kidney events and lower rates of all-cause mortality compared to VKAs (HR: 0.62; 95% CI: 0.43-0.88 and HR: 0.76; 95% CI: 0.59-0.98, respectively).^13^ Interestingly, despite the beneficial effect of rivaroxaban on adverse kidney events and mortality, the authors did not identify any difference between the rivaroxaban and VKA-treated patients regarding the secondary outcomes of net clinical benefit, composite of stroke or other thromboembolic events, and major bleedings.

The beneficial effect of rivaroxaban in lowering the risk of adverse kidney outcomes has not been physiologically explained yet. VKAs have been associated with calcification and injury of renovascular unit, while a potential association of rivaroxaban with reduced vascular inflammation may be associated with this effect.^13^ Either way, rivaroxaban was associated with a delay in CKD progression, with a 61% reduction in the need for chronic kidney replacement treatment when compared to VKAs. In a recent meta-analysis involving trials with AF patients, DOACs were much safer in terms of renal outcomes compared to warfarin.^14^ At a subgroup analysis of rivaroxaban studies, the superiority of rivaroxaban against warfarin in acute kidney injury was verified but was the only renal outcome studied.^14^

Moving forward to a more holistic approach in primary and secondary stroke prevention, as suggested by current guidelines,^15^ among CKD patients with AF, in the equation of thromboembolic and bleeding risk, we have to consider the effect of anticoagulation on kidney function. Although the present study provides reassuring evidence that rivaroxaban exerts beneficial effects on kidney function compared to VKA, still these results are subjected to limitations based on its observational nature. Nevertheless, now we can be a little bit less reluctant when prescribing rivaroxaban to AF patients with severe kidney disease.

## Funding support and author disclosures

Dr Ntaios has received advisory boards/research support/ speaker fees from Abbott, Amgen, AstraZeneca, Bayer, BMS, Boehringer-Ingelheim, Javelin, Novartis, Pfizer, and Sanofi. All other authors have reported that they have no relationships relevant to the contents of this paper to disclose.
